# Cross-Disorder Analysis of De Novo Variants Increases the Power of Prioritising Candidate Genes

**DOI:** 10.3390/life11030233

**Published:** 2021-03-12

**Authors:** Kuokuo Li, Zhengbao Ling, Tengfei Luo, Guihu Zhao, Qiao Zhou, Xiaomeng Wang, Kun Xia, Jinchen Li, Bin Li

**Affiliations:** 1 National Clinical Research Center for Geriatric Disorders, Department of Geriatrics, Xiangya Hospital, Central South University, Changsha 410008, China; likuokuo@sklmg.edu.cn (K.L.); ghzhao@csu.edu.cn (G.Z.); zhouqiao2013@csu.edu.cn (Q.Z.); 2 Department of Obstetrics and Gynecology, The First Affiliated Hospital of Anhui Medical University, No 218 Jixi Road, Hefei 230022, China; 3 Center for Medical Genetics & Hunan Key Laboratory of Medical Genetics, School of Life Sciences, Central South University, Changsha 410008, China; ling2018@csu.edu.cn (Z.L.); ltf2020@csu.edu.cn (T.L.); wangxiaomeng@sklmg.edu.cn (X.W.); xiakun@sklmg.edu.cn (K.X.); 4 Department of Neurology, Xiangya Hospital, Central South University, Changsha 410008, China; 5 Mobile Health Ministry of Education—China Mobile Joint Laboratory, Xiangya Hospital, Central South University, Changsha 410008, China

**Keywords:** neurodevelopmental disorder, de novo variant, candidate gene

## Abstract

De novo variants (DNVs) are critical to the treatment of neurodevelopmental disorders (NDDs). However, effectively identifying candidate genes in small cohorts is challenging in most NDDs because of high genetic heterogeneity. We hypothesised that integrating DNVs from multiple NDDs with genetic similarity can significantly increase the possibility of prioritising the candidate gene. We catalogued 66,186 coding DNVs in 50,028 individuals with nine types of NDDs in cohorts with sizes spanning from 118 to 31,260 from Gene4Denovo database to validate this hypothesis. Interestingly, we found that integrated DNVs can effectively increase the number of prioritised candidate genes for each disorder. We identified 654 candidate genes including 481 shared candidate genes carrying putative functional variants in at least two disorders. Notably, 13.51% (65/481) of shared candidate genes were prioritised only via integrated analysis including 44.62% (29/65) genes validated in recent large cohort studies. Moreover, we estimated that more novel candidate genes will be prioritised with the increase in cohort size, in particular for some disorders with high putative functional DNVs per individual. In conclusion, integrated DNVs may increase the power of prioritising candidate genes, which is important for NDDs with small cohort size.

## 1. Introduction

Neurodevelopmental disorders (NDDs) are disorders with high clinical heterogeneity, leading to considerable personal suffering, morbidity, and disability, which also increase the burden of global healthcare [1]. NDDs, including autism spectrum disorder (ASD), congenital heart disease (CHD), developmental disorders/intellectual disability (DD/ID), epileptic encephalopathy (EE), and schizophrenia (SCZ), are diagnosed following clinical practise guidelines created by practiced experts based on symptoms and signs. However, the diagnosis does not follow a uniform biological framework, which leads to the clinical heterogeneity and overlap between different kinds of NDDs. According to previous studies, almost 26% of patients with ASD, DD, or ID, presented with the clinical phenotypes of EE [2]. Additionally, patients with CHD were reported to share significant clinical features [3,4] and genetic components with those with other NDDs [5]; 10% of CHD cases and 50% of severe CHD cases have a similar clinical phenotype to those with other NDDs [6].

With the development of next-generation sequencing technologies, genetic disruptions have been identified as the major reasons for NDDs, especially for de novo variants (DNVs) with important functional effects contributing to early NDDs. Candidate genes in ASD [7], CHD [8], DD [9], EE [10], ID [11], and SCZ [12] were detected and prioritised successfully using whole-exome sequencing (WES) or whole-genome sequencing (WGS) in recent studies. For instance, due to strong functional effects of DNVs, *CHD8* [13] was found to be associated with ASD, ID, sleeping problems, macrocephaly, and gastrointestinal symptoms, while *DYRK1A* [14] is associated with ID, microcephaly, and febrile seizures infancy. However, for most NDDs, only several candidate genes have been identified based on DNVs due to genetic heterogeneity, rarity DNVs, limited cohort size, and small gene-level relative risks. This genotype-phenotype association method was validated in our studies based on GeneMatcher [15,16,17]. To prioritise additional candidate genes, detect DNVs in specific genes, and perform genotype–phenotype analysis, as well as statistical burden analysis, large cohort size is required. However, sequencing new patients with NDDs requires time and funds, which is not the optimal choice. Another method is to integrate DNVs from multiple NDDs with phenotypic similarities to increase the statistical power of candidate gene discovery, which has been validated recently [18,19,20].

In this study, we aimed to validate the performance of cross-disorder analysis in prioritising candidate genes. We catalogued DNVs in 50,028 individuals with nine types of NDDs that had clinical and genetic similarities. Our results demonstrated that integrating DNVs of different disorders effectively increased the number of prioritised candidate genes. All novel candidate genes shared putative functional variants with more than one disorder. Moreover, we found that both gene-level relative risks and cohort size were the major contributors to candidate gene prioritisation.

## 2. Materials and Methods

### 2.1. Data Collection and Annotation

We collected DNVs detected using WGS or WES in 48 published studies (Table S1). The redundant DNVs were removed based on the description of these studies. We focused on DNVs in nine kinds of NDDs with cohort sizes spanning from 118 to 31,260, including ASD, SCZ, EE, DD/ID, CHD, Tourette disorder (TD), bipolar disorder (BP), obsessive-compulsive disorder (OCD), and complex motor stereotypies (CMS). ANNOVAR [21] and VarCards [22] were used to annotate DNVs in the human reference genome (hg19). We catalogued DNVs into five classes as follows: (1) Loss-of-function (LoF) variant including splicing (≤2 bp), stopgain, and stoploss SNVs, and frameshift indels; (2) deleterious missense (Dmis) variant; (3) tolerant missense (Tmis) variant; (4) synonymous (Syn) variant; (5) non-frameshift indels (NF) variant. We used ReVe [23] to predict Dmis/Tmis variants. Both LoF and Dmis variants were defined as putative functional (Pfun) variants. All these variants are available in our Gene4Denovo database [24].

### 2.2. Overlap of Genes across NDDs Based on De Novo Variants

To test genetic similarities among different NDDs, we used DNENRICH [12,25] software, taking gene size, structure, and local trinucleotide variant rate into consideration to test whether one specific kind of DNVs was significantly shared between any two NDDs. We focused on LoF, Dmis, and Pfun, which increase the genetic risk of NDDs. For this test, we counted the variant number of each gene in each disorder and then calculated the number of overlapping variants between any two disorder. Based on the observed number of variants for each disorder, we randomly selected the matching gene number from all human genes and calculated the expected genetic overlap between any two disorders. Permutation tests were performed to estimate genetic similarity. Details of this method can be found at https://fromem03.u.hpc.mssm.edu/dnenrich/ (accessed on 25 September 2018).

### 2.3. Candidate Genes Prioritization Based on TADA

We next performed transmitted and de novo association (TADA) analysis [26,27] to prioritize candidate genes. In this study, we used TADA-Denovo which identified candidate genes only based on DNMs. TADA-Denovo is a Bayesian model which used observed Pfun DNVs including LoF and deleterious missense variant and expected de novo mutation rate to prioritised candidate gene of disease. Genes carrying significant more Pfun DNVs than expectation were defined as candidate genes. In the first strategy, we counted LoF and Dmis DNVs number in each disorder and performed TADA to calculate the false discovery rate (FDR) for each gene. In the second strategy, LoF and Dmis DNVs in each gene of all NDDs were counted to perform TADA analysis based on the shared genetic components of NDDs. Genes with FDR < 0.05 in these two strategies were defined as candidate genes. Genes carrying Pfun DNVs in more than one disorder were defined as shared genes, and those carrying DNVs in only one disorder were defined as unique genes.

### 2.4. Predicted Gene Discovery Rate

To determine gene discovery rate of each disorder in increased sample size, we sampled (with replacement) populations of 500, 1000, 2000, 4000, 8000, 16,000, and 32,000 cases, and performed TADA analysis to prioritise candidate genes. As TADA only considers Pfun (LoF and Dmis), we used Pfun per individual of each disorder and the sampling number to calculate Pfun DNVs and then performed TADA analysis. The number of genes with FDR < 0.05 of each prediction was counted.

### 2.5. Statistical Analysis

We performed statistical analyses by using R software (v3.5.0) and Linux system (vCentOS 7.1). The R code are available in supplementary file and related input files are available from the corresponding author on reasonable request. The genetic similarity between any two NDDs were performed by DNENRICH software (v1.0). TADA software was used to prioritize candidate gene. *p*-value or FDR in method less than 0.05 were defined as statistically significant. The detail information of two statistical methods were as follows:

DNENRICH (https://fromem03.u.hpc.mssm.edu/dnenrich/) (accessed on 25 September 2018).

TADA (http://www.compgen.pitt.edu/TADA/TADA_guide.html) (accessed on 1 September 2020).

## 3. Results

We curated a total of 348,812 DNVs from 50,028 patients with nine kinds of NDDs with varying sample sizes as reported in 48 published studies (Table 1; Table S1). The DD/ID (*n* = 31,260) accounted for the highest proportion of NDDs in this study and included the highest number of trios based on a WES study to detect coding DNVs (*n* = 44,825) in NDDs. ASD (*n* = 10,318), SCZ (*n* = 3402), CHD (*n* = 2645), EE (*n* = 973), and TD (*n* = 909) were also major NDDs in the identification of candidate genes based on DNVs. However, there was only a small number of patients with BP (*n* = 219), OCD (*n* = 118), and CMS (*n* = 184). In addition, we also found significant Pfun enrichment in NDDs, which were not associated with the sample size (Table 1). DD/ID, OCD, and EE exhibited the most DNVs enrichment, revealing 0.60, 0.58, and 0.55 Pfun DNVs per patient, respectively. Whereas ASD, CHD, SCZ, and TD showed 0.40, 0.39, 0.32, and 0.31 Pfun DNVs per patient, respectively, and exhibited lower DNV enrichment followed by BP (0.25) and CMS (0.21) (Table 1).

**Figure 1:**
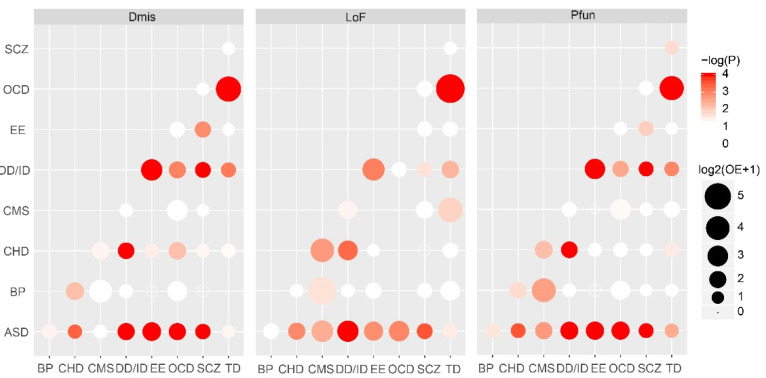
Genetic similarity between different neurodevelopmental disorders. Genetic similarity among disorders were performed based on three classes of variants include LoF, Dmis and Pfun. OE, ratio of observed to expected numbers of shared genes. Solid and coloured circle indicate OE greater than 1 and *p* value less than 0.05. Solid circle with no colour indicate OE greater than 1 but *p* value great than 0.05. Solid circle with no colour indicate OE greater than 1. Hollow circle indicate that OE less than 1. Dmis, Deleterious missense variants; LoF, loss of function. LoF include frameshift, stoploss and stopgain, splicing variants. Pfun, Putative functional variants, including Dmis and LoF variants. *p* value was calculated by using DNENRICH software (v1.0). ASD, autism spectrum disorder; SCZ, schizophrenia; EE, epileptic encephalopathy; DD/ID, developmental disorders/intellectual disability; CHD, congenital heart disease, TD, Tourette disorder; BP, bipolar disorder; OCD, obsessive-compulsive disorder; CMS, complex motor stereotypies.

As LoF, Dmis and Pfun contributed to the formation of NDDs, we explored genetic similarities between any two NDDs using these two kinds of DNVs based on DNENRICH. As nine kinds of NDDs were involved in the genetic similarity analysis, we performed 36 (9 × 8/2) tests. We found that most NDDs showed similar genetic components with others in Dmis, LoF, and Pfun, in particular for ASD and DD/ID. ASD exhibited significant genetic similarity (*p* < 0.05) with 7/8, 7/8, and 8/8 of other NDDs in Dmis, LoF, and Pfun, respectively (Figure 1). DD/ID exhibited significant genetic similarity (*p* < 0.05) with 6/8 of other NDDs in Dmis, LoF, and Pfun, respectively (Figure 1). We did not find statistically significant genetic overlapping among NDDs in 44.44% (16/36), 55.56% (20/36), and 41.67% (15/36) of Dmis-, LoF-, and Pfun-based comparisons, respectively, which might be due to genetic heterogeneity, limited cohort size, or small gene-level relative risks, but 94.4% (34/36) of observed genetic overlapping was high than expected (OE > 1) (Figure 1).

Based on the TADA analysis of Pfun DNVs in nine NDDs, we prioritised 589 candidate genes (FDR < 0.05), containing 104, 8, 24, 527, 22, and 1 genes in ASD, SCZ, EE, DD/ID, CHD, and CMS, respectively (Table 2; Table S2). Due to the smaller number of sample size and low contribution of DNVs, we did not prioritise any candidate gene in TD, BP, and OCD based on DNVs in single disorder. Since most NDDs exhibited more genetic similarities than expected (OE > 1), we integrated Pfun DNVs to all NDDs and performed TADA analysis. We prioritised a total of 523 candidate genes with FDR < 0.05, including 65 novel genes that were not included in the above 589 genes (Table S2). Genes carrying putative functional DNVs in a specific disorder, which passed each FDR threshold in the integration analysis was defined as a candidate gene of this disorder. We found that integrated DNVs increased the number of prioritised candidate genes for each disorder from 5 to 258, in particular for disorders that exhibited more genetic overlapping with integrated DNVs or those with large sample size. This was observed in ASD (*n* = 10,318, *p*-value = 1.00 × 10^−4^, OE = 3.72), SCZ (*n* = 3402, *p*-value = 1.00 × 10^−4^, OE = 1.67), EE (*n* = 973, *p*-value = 1.00 × 10^−4^, OE = 6.54), and CHD (*n* = 2645, *p*-value = 1.00 × 10^−4^, OE = 2.84). Moreover, we prioritised putative candidate genes of NDDs with a small cohort size, including TD, OCD, CMS, and BP. For TD and OCD, we prioritised 28 and 14 novel candidate genes, respectively, compared with zero in a single disorder-based analysis. These two disorders exhibited relatively more genetic similarities with other NDDs and allowed us to prioritise more candidate genes. For CMS and BP, we prioritised nine and five novel candidate genes, respectively, compared with one and zero in a single disorder-based analysis.

We prioritised a total of 654 candidate genes with FDR < 0.05 (Table 3; Table S2). Based on the strength of the statistical evidence, we ranked candidate genes into four ranks as follows: Rank 1 (FDR ≤ 0.0001, *n* = 316); rank 2 (0.0001 < FDR < 0.001, *n* = 60); rank 3 (0.001 < FDR < 0.01, *n* = 101); and rank 4 (0.01 < FDR < 0.05, *n* = 177). Moreover, based on the number of disorders carrying Pfun DNVs of a specific gene, we identified six groups of candidate genes. Precisely 26.45% (173/654) of candidate genes showed Pfun DNMs in only one disorder and 36.54% (239/654), 25.54% (167/654), 9.02% (59/654), 1.99% (13/654), and 0.46% (3/654) of candidate genes showed Pfun DNMs in two, three, four, five, and six disorders, respectively (Table 3). For example, *CACNA1E* (FDR = 1.00 × 10^−11^), *KMT2C* (FDR = 6.31 × 10^−15^), and *KDM5B* (FDR < 2.00 × 10^−18^) showed Pfun in six NDDs. Integrated analysis prioritised 65 novel candidate genes compared to a single disorder-based analysis and all showed Pfun DNVs in more than one disorder (Table S2). To validate these novel candidate genes, we compared them with previously identified candidate genes of NDDs [20,28,29,30,31,32]. In addition, the gene with an expression value > 1 read per kilobase per million map reads in > 50% human brain samples or in >50% human foetal brain samples were defined as a gene expressed in the brain using the BrainSpan database. We found that 44.62% (29/65) of novel candidate genes were reported previously [20,28,29,31,32,33]. Moreover, we used the probability of loss-of-function intolerance (pLI) > 0.9 to filter candidate genes and 55.18% (16/29) passed this threshold, including *SPRY2*, *PSMD12*, *RALA*, *CIC*, *ATP1A1*, *ZMYND8*, *BHLHE40*, *NR6A1*, *RYR2*, *GGNBP2*, *EIF4A2*, *RAB11A*, *CTR9*, *RAB2A*, *UPF3B*, and *KCNC1*. We did not identify additional 55.38% (36/65) novel genes in previous studies [20,28,29,31,32,33]; however, we identified 30.56% (11/36) of the genes with a pLI > 0.9, including *RNF220*, *AP1G1*, *TRIM8*, *LHX2*, *CRIM1*, *UBR3*, *RPSA*, *WDR20*, *SUFU*, *PHEX*, and *KPNA1*.

To strengthen the evidence for candidate genes, we sourced them on a priority basis from genome wide association studies (GWAS) of NDDs based on a threshold of *p* < 10–5 in GWAS Catalog database (https://www.ebi.ac.uk/gwas/ (accessed on 6 March 2021)). We noted that 14.68% (96/654) of candidate genes that were associated with NDDs by common SNPs (Table S2) which was significant than random expectation (Fisher’s exact test, *p* = 0.023, OR = 1.29, 95% CI 1.03–1.61). This result provided bidirectional genetic evidence for these genes.

**Figure 2:**
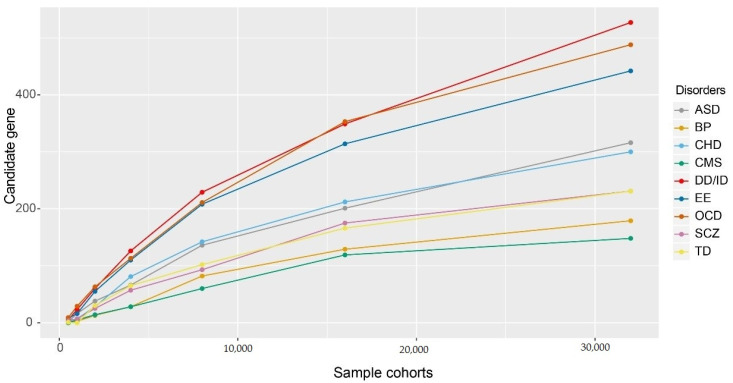
Projected gene discovery in larger cohort size. We assume the sample size were 500, 1000, 2000, 4000, 8000, 16,000, and 32,000 for each disorder and sampling de novo variant from exist based on putative functional de novo variant rate per individual. We then estimate the number of candidate gene for each disorders with FDR < 0.05 by transmitted and de novo association (TADA) analysis. ASD, autism spectrum disorder; SCZ, schizophrenia; EE, epileptic encephalopathy; DD/ID, developmental disorders/intellectual disability; CHD, congenital heart disease, TD, Tourette disorder; BP, bipolar disorder; OCD, obsessive-compulsive disorder; CMS, complex motor stereotypies.

Based on the existing DNVs, we projected the gene discovery rate in an increased sample size. We sampled cohorts with 500, 1000, 2000, 4000, 8000, 16,000, and 32,000 sample sizes for each disorder and performed TADA analysis to prioritise candidate genes. We found that the gene discovery rate rapidly increased with the increasing sample size and then gradually reached a plateau for most NDDs (Figure 2). In addition, we found that putative functional DNVs enrichment was positively correlated with candidate gene discovery rate and negatively correlated with candidate gene discovery plateau. DD/ID, OCD, and EE revealed 0.60, 0.58, and 0.55 putative functional DNVs per patient, respectively, and prioritised more candidate genes with the same cohort size than other disorders. We did not find a candidate gene discovery plateau in the cohort with 32,000 samples. ASD, CHD, SCZ, TD, BP, and CMS exhibited low putative functional DNV enrichment, revealing 0.40, 0.39, 0.32, 0.31, 0.25, and 0.21 Pfun DNVs per patient, respectively. These six NDDs gradually reached a plateau with reduced putative functional DNVs enrichment. For example, BP and CMS plateau in candidate genes were prioritised in the cohort with 16,000 samples.

## 4. Discussion

In this study, we tried to explore a new method to improve the effectiveness of NDD candidate genes by integrating NDDs with similar clinical features. We observed that DD/ID, OCD, and EE exhibited the most DNVs enrichment, revealing 0.60, 0.58, and 0.55 Pfun DNVs per patient, while BP exhibited 0.25 Pfun DNVs per patient, and CMS exhibited 0.21 Pfun DNVs per patient.

NDDs cause suffering, morbidity, and disability, with challenges in the diagnosis and treatment, due to the high clinical heterogeneity within individual disorders [34]. Next-generation sequencing technologies have revealed that DNVs play an important role, functionally contributing to the development of NDDs. However, the genetic heterogeneity, rarity of DNVs, limited cohort size, and small gene-level relative risks, are major challenges for identifying novel candidate genes, which might result in reduced effectiveness in screening for disease-candidate genes [7,8,9,10,11,12]. Due to the limited cohort size, for CMS (*n* = 184), only one candidate gene was prioritised and for BP (*n* = 219), OCD (*n* = 118), and TD (*n* = 909) no candidate genes were prioritised in the single disease-based analysis. It is laborious and costly to collect sufficient cohort information and genomic data, especially for the NDDs with low incidence rates. Therefore, it is a great challenge to study NDDs in-depth with small sample size.

Further analyses of genetic components in the present study showed that NDDs presented similar genetic components in Dmis, LoF, and Pfun. We prioritised a total of 523 candidate genes, including 65 novel genes, which could not be screened by analysing a single NDD. This suggested that integrated analyses could reveal potentially useful data that single disorder-based analysis might have missed. By comparing our findings with previously reported NDD candidate genes and analysing the expression of novel genes using the BrainSpan database, we observed that 29 novel genes were expressed in the brain, overlapping with known candidate genes. Exactly 16 out of 29 genes, including *SPRY2*, *PSMD12*, *RALA*, *CIC*, *ATP1A1*, *ZMYND8*, *BHLHE40*, *NR6A1*, *RYR2*, *GGNBP2*, *EIF4A2*, *RAB11A*, *CTR9*, *RAB2A*, *UPF3B*, and *KCNC1* were more intolerant to LoF with a pLI > 0.9. This suggested that the results of integrated analyses were highly reliable. In other 36 genes, we detected that 11 genes with LoF, such as *RNF220*, *AP1G1*, *TRIM8*, *LHX2*, *CRIM1*, *UBR3*, *RPSA*, *WDR20*, *SUFU*, *PHEX*, and *KPNA1*, were more likely to cause disease.

Compared with previous studies [20,28,29,31,32,33], the 11 genes have not been screened out as the candidate genes of NDDs. Through literature research, we found that most of the 11 novel genes were associated with NDDs. For instance, *RNF220* contributes to noradrenergic neuron development [35] and specifies spinal progenitor domains [36]. *AP1G1* plays an important role in the PI3K/AKT pathway, which is not only associated with cancers but also with megalencephaly [37], ASD [37,38], neurodevelopmental delay [39], and other NDDs [40]. Both *TRIM8* [41] and *UBR3* can act on *CTNNB1*, which can promote the Wnt signalling pathway. Notably, the Wnt signalling pathway is a classical neuron development signalling pathway [42]. Meanwhile, *TRIM8* [43] and *RPSA* [44] are also an important regulators of the PI3K-AKT-mTOR signalling pathway, which is a developmental disease-related signalling pathway [40]. *LHX2* is a regulator of neural differentiation [45,46]. Furthermore, *LHX2* controls cortical size by regulating the balance between proliferation and differentiation in cortical progenitors [45]. Other novel genes identified in this study are associated with cancer, such as *WDR20* with medulloblastoma [47] and *SUFU* with renal cell carcinoma [48,49]. We previously found that NDDs share a common genomic basis with cancers [42]. Therefore, we hypothesised that these genes contribute effectively to the development of NDDs.

Based on projection estimates, we found that gene discovery was affected by sample size and DNV per individual for each disorder. These results were consistent with previous studies. In our future studies, we will prioritise more novel candidate genes and strengthen the genetic evidence of previous putative candidate genes with the increased sample size. For example, Deciphering Developmental Disorders Study identified 94 candidate genes in 4293 families [9] and 285 genes involved in developmental disorders in 31,058 parent-offspring trios [29]. DNVs are also significant influences for candidate gene discovery. For example, based on the sampling method and hypothetical 3000 trios, about 200 high confidence candidate gene (FDR < 0.1) and 300 probable candidate gene (FDR < 0.3) were proposed for the OCD [50] but for the TD only about 25 high confidence candidate genes (FDR < 0.1) and 80 probable candidate genes (FDR < 0.3) were discovered [51]. Moreover, the degree of functional disruption of the variant can also influence the gene discovery rate. Coe et al. found that gene discovery based on LoF and Dmis variants reaches a plateau and may identify a few novel candidate genes. However, increased cohort size will probably identify more novel candidate genes based on fewer severe de novo missense variants, in particular, missense variant cluster in specific hotspot regions, which was not studied previously [20].

DNVs contribute significant to NDDs and one single DNV might result in the formation of NDDs. Previous studies also found than DNV exhibited potential cumulative effect to NDDs which was defined as “oligogenic model” [52,53,54]. Du et al. found that the number of patients with ASD carrying multiple extreme DNVs are significant more than controls. In addition, they also found that patients with ASD carrying more than 2 DNVs exhibited lower IQ than patients carrying 1 or 0 DNV [55]. This was consistent with another study which found that patients with DD/ID carrying more DNVs than ASD [20]. Gifford et al. found three missense variants contribute to heart disease [56]. These results indicated that genes with deleterious variants in one patient might participate in common biological pathway or one variant work as genetic modifier to other genes.

There are still some limitations to this study. First, the large difference in sample sizes of different NDDs may lead to genetic statistical bias, although we statistically corrected these. Second, only classical LoF including classical splicing, stopgain, and stoploss SNVs, and frameshift indels were involved into conventional TADA analysis. Other kinds of variants such as de novo cryptic splice variants predicted by SpliceAI [57], inframe indels variants and small de novo CNV deletions (SmallDel) could also participate in the formation of NDDs. For example, Ruzzo take SmallDel into adjusted TADA analysis [31]. Third, we used in silico tool to predicted missense variant and not all predicted deleterious missense are pathogenic. The combined of multiple tools are useful to identify truly positive candidate genes [58]. Fourth, the novel candidate genes screened in this study by using bioinformatic tools and further functional experimental verification were necessary to validate whether genes were involved in NDDs. Fifth, environmental factors are also involved in the aetiology of NDDs. Combining environmental factors with genetic factors in further research will improve the accuracy and efficiency of DNVs research.

## 5. Conclusions

Taken together, it is inferred from the existing evidence that 11 new candidate genes are relatively reliable for further research. Integrated analysis can effectively improve the candidate gene discovery rate in NDDs. This study provides a new idea for genetic research of NDDs with insufficient samples.

## Figures and Tables

**Table 1:** Summary of collected DNVs in neurodevelopmental disorders.

Phenotypes	Study	Trios	DNVs	Coding DNVs	PTVs	Dmis	Pfun	Pfun per Individual
ASD	14	10,318	287,444	12,141	1580	2507	4087	0.40
SCZa	11	3402	3422	3357	358	716	1074	0.32
EE	9	973	1248	1191	170	364	534	0.55
DD/ID	6	31,260	45,541	44,825	7078	11,683	18,761	0.60
CHD	1	2645	2990	2981	369	654	1023	0.39
TD	3	909	842	818	85	199	284	0.31
BPa	3	219	6995	199	34	21	55	0.25
OCD	1	118	134	128	48	20	68	0.58
CMS	1	184	205	198	27	12	39	0.21

ASD, autism spectrum disorder; SCZ, schizophrenia; EE, epileptic encephalopathy; DD/ID, developmental disorders/intellectual disability; CHD, congenital heart disease, TD, tourette disorder; BP, bipolar disorder; OCD, obsessive-compulsive disorder; CMS, complex motor stereotypies; DNVs, de novo variants; PTVs, protein-truncating variants; Dmis, deleterious missense variant; Pfun, putative functional variant, combining PTVs and Dmis. a, several patients with SCZ/BP come from one study.

**Table 2:** Comparison of prioritised candidate gene number by integrated analysis based on mutation type.

Disorders (N)	Genetic Similarity		Category	Type	FDR < 0.0001	0.0001 < FDR < 0.001	0.001 < FDR < 0.01	0.01 < FDR < 0.05
	*p*-Value	OE						
ASD (10,318)	1.00 × 10^−4^	3.72	Before		24	7	23	50
			After	Pfun	229	31	47	55
				LoF	141	16	24	30
				Dmis	175	21	31	33
SCZ (3402)	1.00 × 10^−4^	1.67	Before		0	0	3	5
			After	Pfun	68	9	17	18
				LoF	29	1	8	13
				Dmis	47	9	11	6
EE (973)	1.00 × 10^−4^	6.54	Before		7	4	5	8
			After	Pfun	87	6	10	6
				LoF	38	1	9	2
				Dmis	58	5	1	5
DD/ID (31,260)	1.00 × 10^−4^	6.80	Before		278	53	81	115
			After	Pfun	287	56	79	96
				LoF	237	46	64	65
				Dmis	267	50	70	73
CHD (2645)	1.00 × 10^−4^	2.84	Before		3	3	4	12
			After	Pfun	78	14	16	20
				LoF	45	6	8	14
				Dmis	46	8	11	10
TD (909)	1.00 × 10^−4^	2.02	Before		0	0	0	0
			After	Pfun	21	1	6	0
				LoF	7	0	2	0
				Dmis	14	1	4	0
BP (219)	2.89 × 10^−2^	1.90	Before		0	0	0	0
			After	Pfun	3	1	1	0
				LoF	2	0	0	0
				Dmis	2	1	1	0
OCD (118)	2.00 × 10^−4^	3.20	Before		0	0	0	0
			After	Pfun	10	1	3	0
				LoF	2	0	0	0
				Dmis	9	1	3	0
CMS (184)	1.00 × 10^−2^	2.49	Before		0	0	0	1
			After	Pfun	4	1	1	3
				LoF	3	0	0	0
				Dmis	1	1	1	3

ASD, autism spectrum disorder; SCZ, schizophrenia; DD/ID, developmental disorders/intellectual disability; CHD, congenital heart disease; TD, Tourette disorder; BP, bipolar disorder; OCD, obsessive-compulsive disorder; CMS, complex motor stereotypies; Pfun, putative functional variant; LoF, loss of function variant; Dmis, deleterious missense variant; Before, prioritised candidate gene base on putative functional DNVs of specific disorder with FDR < 0.05; After, prioritised candidate gene base on the integration of DNVs in all disorders. Gene carrying Pfun, LoF and Dmis in specific disorder and pass each FDR threshold in integration analysis was defined as candidate gene of this disorder. OE, ratio of observed to expected numbers of shared genes with putative functional de novo variants.

**Table 3:** Candidate gene carrying putative functional variants in different number of disorders (FDR < 0.05).

Rank (FDR)	Unique Disorders *n* = 173, 26.45%	Two Disorders *n* = 239, 36.54%	Three Disorders *n* = 167, 25.54%	Four Disorders *n* = 59, 9.02%	Five Disorders *n* = 13, 1.99%	Six Disorders *n* = 3, 0.46%
[0, 0.0001) (48.32%)	42	113	98	50	10	3
[0.0001, 0.001) (9.17%)	14	26	18	2	0	0
[0.001, 0.01) (15.44%)	31	41	21	6	2	0
[0.01, 0.05) (27.06%)	86	59	30	1	1	0

Candidate genes are split into six parts based on the number of disorders with putative functional DNMs in specific gene. Unique genes means gene only carry putative functional DNMs in one disorder. We ranked all candidate genes into four tiers based on the strength of FDR.

## Data Availability

All the data in this study can be download from Gene4Denovo database (http://genemed.tech/gene4denovo/home (accessed on 6 March 2021)).

## Supplementary Materials

The following are available online at https://www.mdpi.com/2075-1729/11/3/233/s1, Table S1: WES or WGS studies regarding to DNVs, Table S2: Candidate gene with FDR < 0.05 in this study.
