# Supplementary material for: Cross-Disorder Analysis of De Novo Variants Increases the Power of Prioritising Candidate Genes
Source: Life (Basel). 2021 Mar 12;11(3):233. doi: 10.3390/life11030233 (PMC8001830; doi:10.3390/life11030233)
Supplement: Supplementary file 1 [file life-11-00233-s001.zip › life-1110493-supplementary/Table S1.docx]

**Table S1. WES or WGS studies regarding to DNVs.**

| **Reference** | **PubMed ID** | **Method** | **Samples** | **DNMs** | **Exonic DNMs** |
| --- | --- | --- | --- | --- | --- |
| **Autism spectrum disorder (ASD)** | | | | | |
| Augustine Kong et al. Nature 2012 (1) | 22914163 | WGS | 78 | 4,932 | 74 |
| Jacob J. Michaelson et al. Cell 2012(2) | 23260136 | WGS | 10 | 581 | 10 |
| ASC: Satterstrom et al. Cell (2020)(3) | 31981491 | WES | 3899 | 7473 | 4,728 |
| SSC: Ivan Iossifov et al. Nature 2014(4)  Joon-Yong An et al. Science 2018(5) | 25363768  30545852 | WES  WGS | 2,631 | 130,110 | 3,510 |
| Ryota Hashimoto et al. J Hum Genet. 2016(6) | 26582266 | WES | 30 | 38 | 35 |
| Jinchen Li, et al. Molecular Psychiatry 2017(7) | 28831199 | WGS | 32 | 2,091 | 32 |
| Huidan Wu et al. Clinical genetics. 2020(8) | 31674007 | WES | 67 | 115 | 115 |
| Hui Guo, et al. Genetic in Medicine. 2019(9) | 30504930 | WES/WGS | 200 | 295 | 295 |
| Pamela Feliciano et al. NPJ Genom Med. 2019(10) | 31452935 | WES | 465 | 658 | 647 |
| Elizabeth K. Ruzzo et al. Cell 2020(11) | 31398340 | WGS | 960 | 198 | 197 |
| Jian Jiao et al. JMN, 2019(12) | 31838722 | WES | 59 | 75 | 68 |
| Ryan KC Yuen et al. Nature Neuroscience 2017(13) | 28263302 | WGS | 1,625 | 140,556 | 2,111 |
| Atsushi Takata, et al. Cell Reports 2018(14) | 29346770 | WES | 262 | 322 | 319 |
|  |  |  | **10,318** | **287,444** | **12,141** |
| **Schizophrenia (SCZ)** | | | | | |
| Simon L Girard et al. Nat. Genet. 2011(15) | 21743468 | WES | 14 | 15 | 15 |
| Bin Xu et al. Nat. Genet. 2012(16)  Atsushi Takata et al. Neuron. 2014(17) | 23042115  24853937 | WES | 231 | 180 | 170 |
| Suleyman Gulsuner et al. Cell 2013(18) | 23911319 | WES | 105 | 100 | 100 |
| Menachem Fromer et al. Nature 2014(19) | 24463507 | WES | 617 | 640 | 637 |
| SE McCarthy et al. Molecular Psychiatry 2014(20) | 24776741 | WES | 57 | 65 | 65 |
| Michel Guipponi et al. PLoS ONE 2014(21) | 25420024 | WES | 53 | 49 | 49 |
| Amirthagowri Ambalavanan et al. Eur J Hum Genet. 2016(22) | 26508570 | WES | 17 | 20 | 20 |
| Elliott Rees et al. Nature neuroscience 2020(23) | 31932766 | WES | 613 | 606 | 578 |
| Daniel P. Howrigan et al. Nature neuroscience 2020(24) | 31932770 | WES | 1695 | 1747 | 1711 |
| Claudio Toma et al, JAMA network, 2020(25) | 32383744 | WES/ WGS | 14 | 14 | 12 |
|  |  |  | **3,402** | **3422** | **3,357** |
| **Intellectual disability (ID)** |  |  |  |  |  |
| Vissers LE et al. Nature genetics 2011(26) | 21076407 | WES | 10 | 9 | 9 |
| Anita Rauch et al. Lancet 2012(27) | 23020937 | WES | 51 | 87 | 85 |
| de Ligt J et al. N Engl J Med. 2012(28)  Gilissen C et al. Nature. 2014(29) | 23033978  24896178 | WES,  WGS | 100 | 143 | 141 |
| Fadi F. Hamdan et al. PLoS Genetics 2014(30) | 25356899 | WES | 41 | 81 | 80 |
| Joanna Kaplanis et al. bioRxiv 2020(31) | NA | WES | 31058 | 45,221 | 44,510 |
|  |  |  | **31,260** | **45,541** | **44,825** |
| **Epileptic encephalopathie (EE)** | | | | | |
| Veeramah KR et al. Epilepsia 2013(32) | 23647072 | WES | 10 | 15 | 15 |
| Epi4K Consortium. Nature 2013(33) | 23934111 | WES | 264 | 329 | 293 |
| Appenzeller S et al. Am J Hum Genet. 2014(34) | 25262651 | WES | 92 | 123 | 123 |
| Fadi F. Hamdan et al. Am J Hum Genet. 2017(35) | 29100083 | WGS/WES | 197 | 294 | 294 |
| Helbig KL et al. Genetics in Medicine 2016(36) | 26795593 | WES | 216 | 302 | 291 |
| Henrike O. Heyne et al. Nature genetics 2018(37) | 29942082 | WES | 144 | 140 | 138 |
| Tran Mau-Them F et al. Genetic in medcine 2019(38) | 30166628 | WES | 10 | 10 | 10 |
| John K.L. Wong et al, Neurol Genet. 2018(39) | 29904720 | WES | 23 | 18 | 18 |
| Jinliang Li et al, Scientific report, 2019(40) | 31784560 | WES | 10 | 10 | 9 |
|  |  |  | **973** | **1,248** | **1,191** |
| **Congenital heart disease (CHD)** | | | | | |
| Sheng Chih Jin et al. Nature Genetics 2017(41) | 28991257 | WES | **2,645** | **2,990** | **2,981** |
| **Tourette Disorder (TD)** |  |  |  |  |  |
| Sheng Wang et al. Cell Reports 2018(42) | 30257206 | WES | 802 | 775 | 773 |
| Am J Med Genet B Neuropsychiatr Genet. 2017(43) | 28608572 | WES | 10 | 30 | 8 |
| Shiguo Liu et al. Mol Psychiatry 2019(44) | 31673123 | WES | 97 | 37 | 37 |
|  |  |  | **909** | **842** | **818** |
| **Bipolar disorder (BP)** |  |  |  |  |  |
| Kataoka M et al. Mol Psychiatry. 2016(45) | 27217147 | WES | 79 | 71 | 68 |
| Fernando S. Goes et al. Mol Psychiatry. 2019(46) | 31776463 | WGS | 97 | 6882 | 107 |
| Claudio Toma et al, JAMA network, 2020(25) | 32383744 | WES/ WGS | 29 | 28 | 24 |
|  |  |  | **219** | **6,995** | **199** |
| **Complex motor stereotypies (CMS)** |  |  |  |  |  |
| Thomas V. Fernandez et al. bioRxiv 2019(47) | NA | WES | **118** | **134** | **128** |
| **Obsessive-Compulsive Disorder (OCD)** |  |  |  |  |  |
| Carolina Cappi et al. Biological Psychiatry 2019(48) | 31771860 | WES | **184** | **205** | **198** |

**Reference**

1. Kong, A., Frigge, M.L., Masson, G., Besenbacher, S., Sulem, P., Magnusson, G., Gudjonsson, S.A., Sigurdsson, A., Jonasdottir, A., Jonasdottir, A. *et al.* (2012) Rate of de novo mutations and the importance of father's age to disease risk. *Nature*, **488**, 471-475.

2. Michaelson, J.J., Shi, Y., Gujral, M., Zheng, H., Malhotra, D., Jin, X., Jian, M., Liu, G., Greer, D., Bhandari, A. *et al.* (2012) Whole-genome sequencing in autism identifies hot spots for de novo germline mutation. *Cell*, **151**, 1431-1442.

3. Satterstrom, F.K., Kosmicki, J.A., Wang, J., Breen, M.S., De Rubeis, S., An, J.Y., Peng, M., Collins, R., Grove, J., Klei, L. *et al.* (2020) Large-Scale Exome Sequencing Study Implicates Both Developmental and Functional Changes in the Neurobiology of Autism. *Cell*, **180**, 568-584 e523.

4. Iossifov, I., O'Roak, B.J., Sanders, S.J., Ronemus, M., Krumm, N., Levy, D., Stessman, H.A., Witherspoon, K.T., Vives, L., Patterson, K.E. *et al.* (2014) The contribution of de novo coding mutations to autism spectrum disorder. *Nature*, **515**, 216-221.

5. An, J.Y., Lin, K., Zhu, L., Werling, D.M., Dong, S., Brand, H., Wang, H.Z., Zhao, X., Schwartz, G.B., Collins, R.L. *et al.* (2018) Genome-wide de novo risk score implicates promoter variation in autism spectrum disorder. *Science*, **362**.

6. Hashimoto, R., Nakazawa, T., Tsurusaki, Y., Yasuda, Y., Nagayasu, K., Matsumura, K., Kawashima, H., Yamamori, H., Fujimoto, M., Ohi, K. *et al.* (2016) Whole-exome sequencing and neurite outgrowth analysis in autism spectrum disorder. *J Hum Genet*, **61**, 199-206.

7. Li, J., Wang, L., Guo, H., Shi, L., Zhang, K., Tang, M., Hu, S., Dong, S., Liu, Y., Wang, T. *et al.* (2017) Targeted sequencing and functional analysis reveal brain-size-related genes and their networks in autism spectrum disorders. *Molecular psychiatry*, **22**, 1282-1290.

8. Wu, H., Li, H., Bai, T., Han, L., Ou, J., Xun, G., Zhang, Y., Wang, Y., Duan, G., Zhao, N. *et al.* (2020) Phenotype-to-genotype approach reveals head-circumference-associated genes in an autism spectrum disorder cohort. *Clin Genet*, **97**, 338-346.

9. Guo, H., Duyzend, M.H., Coe, B.P., Baker, C., Hoekzema, K., Gerdts, J., Turner, T.N., Zody, M.C., Beighley, J.S., Murali, S.C. *et al.* (2019) Genome sequencing identifies multiple deleterious variants in autism patients with more severe phenotypes. *Genetics in medicine : official journal of the American College of Medical Genetics*, **21**, 1611-1620.

10. Feliciano, P., Zhou, X., Astrovskaya, I., Turner, T.N., Wang, T., Brueggeman, L., Barnard, R., Hsieh, A., Snyder, L.G., Muzny, D.M. *et al.* (2019) Exome sequencing of 457 autism families recruited online provides evidence for autism risk genes. *NPJ genomic medicine*, **4**, 19.

11. Ruzzo, E.K., Perez-Cano, L., Jung, J.Y., Wang, L.K., Kashef-Haghighi, D., Hartl, C., Singh, C., Xu, J., Hoekstra, J.N., Leventhal, O. *et al.* (2019) Inherited and De Novo Genetic Risk for Autism Impacts Shared Networks. *Cell*, **178**, 850-866 e826.

12. Jiao, J., Zhang, M., Yang, P., Huang, Y., Hu, X., Cai, J., Yang, C., Situ, M., Zhang, H., Fu, L. *et al.* (2020) Identification of De Novo JAK2 and MAPK7 Mutations Related to Autism Spectrum Disorder Using Whole-Exome Sequencing in a Chinese Child and Adolescent Trio-Based Sample. *J Mol Neurosci*, **70**, 219-229.

13. RK, C.Y., Merico, D., Bookman, M., J, L.H., Thiruvahindrapuram, B., Patel, R.V., Whitney, J., Deflaux, N., Bingham, J., Wang, Z. *et al.* (2017) Whole genome sequencing resource identifies 18 new candidate genes for autism spectrum disorder. *Nature neuroscience*, **20**, 602-611.

14. Takata, A., Miyake, N., Tsurusaki, Y., Fukai, R., Miyatake, S., Koshimizu, E., Kushima, I., Okada, T., Morikawa, M., Uno, Y. *et al.* (2018) Integrative Analyses of De Novo Mutations Provide Deeper Biological Insights into Autism Spectrum Disorder. *Cell reports*, **22**, 734-747.

15. Girard, S.L., Gauthier, J., Noreau, A., Xiong, L., Zhou, S., Jouan, L., Dionne-Laporte, A., Spiegelman, D., Henrion, E., Diallo, O. *et al.* (2011) Increased exonic de novo mutation rate in individuals with schizophrenia. *Nature genetics*, **43**, 860-863.

16. Xu, B., Ionita-Laza, I., Roos, J.L., Boone, B., Woodrick, S., Sun, Y., Levy, S., Gogos, J.A. and Karayiorgou, M. (2012) De novo gene mutations highlight patterns of genetic and neural complexity in schizophrenia. *Nature genetics*, **44**, 1365-1369.

17. Takata, A., Xu, B., Ionita-Laza, I., Roos, J.L., Gogos, J.A. and Karayiorgou, M. (2014) Loss-of-function variants in schizophrenia risk and SETD1A as a candidate susceptibility gene. *Neuron*, **82**, 773-780.

18. Gulsuner, S., Walsh, T., Watts, A.C., Lee, M.K., Thornton, A.M., Casadei, S., Rippey, C., Shahin, H., Consortium on the Genetics of, S., Group, P.S. *et al.* (2013) Spatial and temporal mapping of de novo mutations in schizophrenia to a fetal prefrontal cortical network. *Cell*, **154**, 518-529.

19. Fromer, M., Pocklington, A.J., Kavanagh, D.H., Williams, H.J., Dwyer, S., Gormley, P., Georgieva, L., Rees, E., Palta, P., Ruderfer, D.M. *et al.* (2014) De novo mutations in schizophrenia implicate synaptic networks. *Nature*, **506**, 179-184.

20. McCarthy, S.E., Gillis, J., Kramer, M., Lihm, J., Yoon, S., Berstein, Y., Mistry, M., Pavlidis, P., Solomon, R., Ghiban, E. *et al.* (2014) De novo mutations in schizophrenia implicate chromatin remodeling and support a genetic overlap with autism and intellectual disability. *Molecular psychiatry*, **19**, 652-658.

21. Guipponi, M., Santoni, F.A., Setola, V., Gehrig, C., Rotharmel, M., Cuenca, M., Guillin, O., Dikeos, D., Georgantopoulos, G., Papadimitriou, G. *et al.* (2014) Exome sequencing in 53 sporadic cases of schizophrenia identifies 18 putative candidate genes. *PloS one*, **9**, e112745.

22. Ambalavanan, A., Girard, S.L., Ahn, K., Zhou, S., Dionne-Laporte, A., Spiegelman, D., Bourassa, C.V., Gauthier, J., Hamdan, F.F., Xiong, L. *et al.* (2016) De novo variants in sporadic cases of childhood onset schizophrenia. *European journal of human genetics : EJHG*, **24**, 944-948.

23. Rees, E., Han, J., Morgan, J., Carrera, N., Escott-Price, V., Pocklington, A.J., Duffield, M., Hall, L.S., Legge, S.E., Pardinas, A.F. *et al.* (2020) De novo mutations identified by exome sequencing implicate rare missense variants in SLC6A1 in schizophrenia. *Nature neuroscience*, **23**, 179-184.

24. Howrigan, D.P., Rose, S.A., Samocha, K.E., Fromer, M., Cerrato, F., Chen, W.J., Churchhouse, C., Chambert, K., Chandler, S.D., Daly, M.J. *et al.* (2020) Exome sequencing in schizophrenia-affected parent-offspring trios reveals risk conferred by protein-coding de novo mutations. *Nature neuroscience*, **23**, 185-193.

25. Toma, C., Shaw, A.D., Overs, B.J., Mitchell, P.B., Schofield, P.R., Cooper, A.A. and Fullerton, J.M. (2020) De Novo Gene Variants and Familial Bipolar Disorder. *JAMA Netw Open*, **3**, e203382.

26. Vissers, L.E., de Ligt, J., Gilissen, C., Janssen, I., Steehouwer, M., de Vries, P., van Lier, B., Arts, P., Wieskamp, N., del Rosario, M. *et al.* (2010) A de novo paradigm for mental retardation. *Nature genetics*, **42**, 1109-1112.

27. Rauch, A., Wieczorek, D., Graf, E., Wieland, T., Endele, S., Schwarzmayr, T., Albrecht, B., Bartholdi, D., Beygo, J., Di Donato, N. *et al.* (2012) Range of genetic mutations associated with severe non-syndromic sporadic intellectual disability: an exome sequencing study. *Lancet*, **380**, 1674-1682.

28. de Ligt, J., Willemsen, M.H., van Bon, B.W., Kleefstra, T., Yntema, H.G., Kroes, T., Vulto-van Silfhout, A.T., Koolen, D.A., de Vries, P., Gilissen, C. *et al.* (2012) Diagnostic exome sequencing in persons with severe intellectual disability. *The New England journal of medicine*, **367**, 1921-1929.

29. Gilissen, C., Hehir-Kwa, J.Y., Thung, D.T., van de Vorst, M., van Bon, B.W., Willemsen, M.H., Kwint, M., Janssen, I.M., Hoischen, A., Schenck, A. *et al.* (2014) Genome sequencing identifies major causes of severe intellectual disability. *Nature*, **511**, 344-347.

30. Hamdan, F.F., Srour, M., Capo-Chichi, J.M., Daoud, H., Nassif, C., Patry, L., Massicotte, C., Ambalavanan, A., Spiegelman, D., Diallo, O. *et al.* (2014) De novo mutations in moderate or severe intellectual disability. *PLoS genetics*, **10**, e1004772.

31. Kaplanis, J., Samocha, K.E., Wiel, L., Zhang, Z., Arvai, K.J., Eberhardt, R.Y., Gallone, G., Lelieveld, S.H., Martin, H.C., McRae, J.F. *et al.* (2020) Integrating healthcare and research genetic data empowers the discovery of 28 novel developmental disorders. *bioRxiv*, 797787.

32. Veeramah, K.R., Johnstone, L., Karafet, T.M., Wolf, D., Sprissler, R., Salogiannis, J., Barth-Maron, A., Greenberg, M.E., Stuhlmann, T., Weinert, S. *et al.* (2013) Exome sequencing reveals new causal mutations in children with epileptic encephalopathies. *Epilepsia*, **54**, 1270-1281.

33. Epi, K.C., Epilepsy Phenome/Genome, P., Allen, A.S., Berkovic, S.F., Cossette, P., Delanty, N., Dlugos, D., Eichler, E.E., Epstein, M.P., Glauser, T. *et al.* (2013) De novo mutations in epileptic encephalopathies. *Nature*, **501**, 217-221.

34. Euro, E.-R.E.S.C., Epilepsy Phenome/Genome, P. and Epi, K.C. (2014) De novo mutations in synaptic transmission genes including DNM1 cause epileptic encephalopathies. *American journal of human genetics*, **95**, 360-370.

35. Hamdan, F.F., Myers, C.T., Cossette, P., Lemay, P., Spiegelman, D., Laporte, A.D., Nassif, C., Diallo, O., Monlong, J., Cadieux-Dion, M. *et al.* (2017) High Rate of Recurrent De Novo Mutations in Developmental and Epileptic Encephalopathies. *American journal of human genetics*, **101**, 664-685.

36. Helbig, K.L., Farwell Hagman, K.D., Shinde, D.N., Mroske, C., Powis, Z., Li, S., Tang, S. and Helbig, I. (2016) Diagnostic exome sequencing provides a molecular diagnosis for a significant proportion of patients with epilepsy. *Genetics in medicine : official journal of the American College of Medical Genetics*, **18**, 898-905.

37. Heyne, H.O., Singh, T., Stamberger, H., Abou Jamra, R., Caglayan, H., Craiu, D., De Jonghe, P., Guerrini, R., Helbig, K.L., Koeleman, B.P.C. *et al.* (2018) De novo variants in neurodevelopmental disorders with epilepsy. *Nature genetics*, **50**, 1048-1053.

38. Tran Mau-Them, F., Guibaud, L., Duplomb, L., Keren, B., Lindstrom, K., Marey, I., Mochel, F., van den Boogaard, M.J., Oegema, R., Nava, C. *et al.* (2019) De novo truncating variants in the intronless IRF2BPL are responsible for developmental epileptic encephalopathy. *Genetics in medicine : official journal of the American College of Medical Genetics*, **21**, 1008-1014.

39. Wong, J.K.L., Gui, H., Kwok, M., Ng, P.W., Lui, C.H.T., Baum, L., Sham, P.C., Kwan, P. and Cherny, S.S. (2018) Rare variants and de novo variants in mesial temporal lobe epilepsy with hippocampal sclerosis. *Neurol Genet*, **4**, e245.

40. Li, J., Gao, K., Cai, S., Liu, Y., Wang, Y., Huang, S., Zha, J., Hu, W., Yu, S., Yang, Z. *et al.* (2019) Germline de novo variants in CSNK2B in Chinese patients with epilepsy. *Scientific reports*, **9**, 17909.

41. Jin, S.C., Homsy, J., Zaidi, S., Lu, Q., Morton, S., DePalma, S.R., Zeng, X., Qi, H., Chang, W., Sierant, M.C. *et al.* (2017) Contribution of rare inherited and de novo variants in 2,871 congenital heart disease probands. *Nat Genet*, **49**, 1593-1601.

42. Wang, S., Mandell, J.D., Kumar, Y., Sun, N., Morris, M.T., Arbelaez, J., Nasello, C., Dong, S., Duhn, C., Zhao, X. *et al.* (2018) De Novo Sequence and Copy Number Variants Are Strongly Associated with Tourette Disorder and Implicate Cell Polarity in Pathogenesis. *Cell reports*, **24**, 3441-3454 e3412.

43. Eriguchi, Y., Kuwabara, H., Inai, A., Kawakubo, Y., Nishimura, F., Kakiuchi, C., Tochigi, M., Ohashi, J., Aoki, N., Kato, K. *et al.* (2017) Identification of candidate genes involved in the etiology of sporadic Tourette syndrome by exome sequencing. *American journal of medical genetics. Part B, Neuropsychiatric genetics : the official publication of the International Society of Psychiatric Genetics*, **174**, 712-723.

44. Liu, S., Tian, M., He, F., Li, J., Xie, H., Liu, W., Zhang, Y., Zhang, R., Yi, M., Che, F. *et al.* (2020) Mutations in ASH1L confer susceptibility to Tourette syndrome. *Molecular psychiatry*, **25**, 476-490.

45. Kataoka, M., Matoba, N., Sawada, T., Kazuno, A.A., Ishiwata, M., Fujii, K., Matsuo, K., Takata, A. and Kato, T. (2016) Exome sequencing for bipolar disorder points to roles of de novo loss-of-function and protein-altering mutations. *Molecular psychiatry*, **21**, 885-893.

46. Goes, F.S., Pirooznia, M., Tehan, M., Zandi, P.P., McGrath, J., Wolyniec, P., Nestadt, G. and Pulver, A.E. (2019) De novo variation in bipolar disorder. *Molecular psychiatry*.

47. Fernandez, T.V., Williams, Z.P., Kline, T., Rajendran, S., Augustine, F., Wright, N., Sullivan, C.A.W., Olfson, E., Abdallah, S.B., Liu, W. *et al.* (2019) Primary complex motor stereotypies are associated with de novo damaging DNA coding mutations that identify candidate risk genes and biological pathways. *bioRxiv*, 730952.

48. Cappi, C., Oliphant, M.E., Peter, Z., Zai, G., Conceicao do Rosario, M., Sullivan, C.A.W., Gupta, A.R., Hoffman, E.J., Virdee, M., Olfson, E. *et al.* (2020) De Novo Damaging DNA Coding Mutations Are Associated With Obsessive-Compulsive Disorder and Overlap With Tourette's Disorder and Autism. *Biological psychiatry*, **87**, 1035-1044.
